# Effect of glutamine supplementation and replacement of tris-egg yolk based extender with defatted cow milk on spermatozoa quality after equilibration and thawing

**DOI:** 10.14202/vetworld.2015.1027-1031

**Published:** 2015-08-26

**Authors:** Vasundhara Dawra, Brijesh Yadav, Sarvajeet Yadav

**Affiliations:** Department of Veterinary Physiology, College of Veterinary Science and Animal Husbandry, Pt. Deen Dayal Upadhyaya Pashu Chikitsa Vishwavidyalaya Evam Go Anusndhan Sansthan, Mathura, Uttar Pradesh, India

**Keywords:** Bhadawari bull, defatted milk, glutamine, post-thaw spermatozoa quality

## Abstract

**Aim:**

The present study was designed to evaluate the effect of supplementation of glutamine and replacement of Tris-egg yolk (TE) based buffer with defatted cow milk on the spermatozoa quality after equilibration and thawing.

**Materials and Methods:**

Semen was collected from five Bhadawari bulls biweekly, and a total of 30 ejaculates were taken. The semen ejaculates were pooled and divided into three equal parts. The pooled semen was diluted by TE based extender (control), TE + glutamine (8 mM) (T1) and 50% TE + 50% deffated cow milk + glutamine (8 mM) (T2). At two stages *viz*. after equilibration and after 12 h of cryopreservation (thawed samples), progressive motility, percent live, and percent acrosomal damage of the spermatozoa was assessed.

**Results:**

Supplementation of glutamine improved (p<0.05) the spermatozoa quality with respect to the progressive motility, percent live and acrosomal damage both post-equilibration and post-thaw. T2 improved (p<0.05) the spermatozoa quality as compared to control, however; it was less (p<0.05) effective as compared T1 both post-equilibration and post-thaw.

**Conclusion:**

From the results of present study it can be concluded that glutamine supplementation was effective in maintaining post-equilibration and post-thaw spermatozoa quality whereas defatted cow milk was not as effective as TE based buffer in the extender in improving the spermatozoa quality.

## Introduction

The domestic buffalo is a peculiar species of the Bovidae family. The buffalo population is increasing progressively at over 199 million [1]. More than 95% of the population is located in Asia, where buffaloes play an important role in rural livelihood and nutritional security. In recent decades, buffalo population is burgeoning in different parts of the world and India in particular with the rate of more than 5% [2].

Artificial insemination (AI) has proved to be a game changer in augmenting livestock production potential; however, the quality of frozen-thawed semen is the biggest challenge to the scientific fraternity which affects the conception rate [3]. Application of AI with frozen-thawed semen in buffalo has been reported to be less successful because of poor freezability and fertility as compared with cattle [4,5]. Hence, successful cryopreservation of buffalo semen would help in storage of male gametes and the maintenance of genetic stock that could exploit the production associated with the economic value of buffalo.

Cryopreservation is a non-physiological method that involves acclimatization of spermatozoa to thermal, osmotic and myriad biochemical changes during the dilution, cooling-freezing and thawing processes [6]. Damage occurring during the freezing-thawing procedures affects mainly the plasma membrane, acrosome integrity, mitochondria and nucleus [6,7]. Therefore, such changes in the integrity of spermatozoa affect the fertilizability. Conception rate in buffaloes inseminated with liquid semen is reported to be 60% [8] however, with frozen-thawed semen under field conditions it is approximately 30% [9,10]. The gap in the conception rate using liquid semen and frozen-thawed buffalo semen indicates that cryopreservation adversely affects the viability and the fertilizing potential of buffalo bull spermatozoa. Therefore, there is a need to minimize the damage of spermatozoa during cryopreservation.

The buffalo spermatozoa are more susceptible to hazards during freezing and thawing [5] because of higher lipid ratio of the spermatozoa [11] which contributes to more production of free radicals. Amino acids protect the structural architecture of biological membranes of sperm cells during the cryopreservation and thawing process [12]. Glutamate is formed in a substantial amount of seminiferous tubules [13] and constitutes 90% of total amino acids of rete-testes fluid [14]. Amongst the amino acids used in freezing extender, glutamine/glutamic acid has been reported to be used as an additive in several species such as angora goat [15], ram [16], donkey [17], rabbit [18] and bull [19,20] spermatozoa against freeze-thawing damage. The cryoprotective nature of glutamine is attributed to its anti-oxidative property which is responsible for improved post-thaw motility pattern and plasma membrane integrity of spermatozoa [20]. Defatted cow milk was reported to be used as diluents for the storage of liquid semen [21].

The present study was conducted to investigate the effect of supplementation of glutamine and replacement of Tris-egg yolk (TE) based buffer with defatted cow milk on the spermatozoa quality post-equilibration and post-thaw.

## Materials and Methods

### Ethical approval

The ethical approval was not required to pursue this research work. However semen from each bull was collected as per standard collection procedure without harming or giving stress to the animal.

### Experimental location

The experiment was carried out at the district dairy demonstration farm of the College of Veterinary Sciences and Animal Husbandry, Uttar Pradesh Pandit Deen Dayal Upadhayaya Pashu Chikitsa Vigyan Vishwavidyalaya Evam Go Anusandhan Sansthan, Mathura, Uttar Pradesh, India which is located in the semiarid region of the country at longitude 78°E and latitude 27°N and at an altitude of 176 m above mean sea level.

### Experimental animals and management

Five healthy adults, Bhadawari buffalo bulls (aged, 3.76±0.34 years and weight 416.32±16.76 kg) were used as semen donors. The animals were provided balanced ration as per the recommendations of national research council [22] and clean drinking water *ad-libitum*. Prophylactic measures against foot and mouth disease, hemorrhagic septicemia, endo- and ecto-parasitic infestations were carried out as prescribed by the health calendar of the farm to ensure that the animals were in a healthy condition throughout the study. All the experimental bulls were housed in individual pens made up of brick and concrete floor and asbestos roof with the slopes toward the drain to avoid dampness.

### Experimental design

The experiment was carried out during the month of February and March when the environmental temperature was under thermoneutral zone to avoid any seasonal variation in seminal attributes. Semen was collected from five Bhadawari bulls biweekly from each bull with the help of artificial vagina (AV) on dummy animal between 8.00 and 9.00 h using a standard protocol. A total of 30 ejaculates were taken for five bulls. In fresh ejaculates, the physical properties of the semen, mass motility, progressive motility, concentration, and percent live spermatozoa were examined. The qualified ejaculates were pooled and divided into three equal parts. The pooled semen was diluted by TE based extender (conrol), TE + glutamine (8 mM) (T1) and 50% TE + 50% defatted milk + glutamine (8 mM) (T2). The semen was diluted to obtain a final concentration of approximately 80-100 million spermatozoa/ml. The diluted semen was equilibrated and cryopreserved. At two stages *viz*. after equilibration and after 12 h of cryopreservation (thawed samples) progressive motility, percent live and percent acrosomal damage of spermatozoa was assessed.

### Collection of semen

All the buffalo bulls were trained for semen donation before beginning the experiment. Prepuces of the bulls were washed before the semen collection with 0.01% acriflavine solution. Semen was collected into a dry, clean graduated tube attached to the latex cone of the AV. After collection, the semen samples were immediately transferred to a water bath maintained at 35°C and exposure to direct sunlight, and bacterial contamination was avoided.

### Preparation of defatted cow milk

The defatted cow milk was prepared as per the described procedure [21]. The milk was heated up to 90°C for 10-12 min and then cooled overnight in a refrigerator. The fat layer was removed, and the milk was reheated in a water bath for 10-12 min. After repeated cooling, the remaining fat was removed by filtration through cotton wool.

### Glycerization, equilibration and cryopreservation

3% of glycerol was added to extended semen at room temperature and incubated for 2 h at 15°C. After 2 h, equal volume of pre-cooled (10-15°C) 11% glycerol was added 3 times at the interval of 15 min to achieve a spermatozoa concentration of 80-100 million/ml. The final volume of extended semen was equilibrated for 90 min at 5°C in cool handling cabinet. After equilibration, the semen was aspirated into French mini straws, sealed with polyvinyl alcohol powder and cooled horizontally from 5°C to −140°C in liquid nitrogen vapor for 10 min duration and plunged into liquid nitrogen for storage.

### Semen analysis

The volume of the semen was measured directly by observing the level of the semen in the graduated tube. The mass motility of the ejaculated samples was evaluated by observing the waves pattern at 1-5 scale [23]. The progressive motility was determined by placing 5 μl of diluted semen on a warmed glass slide (37°C) and allowed to spread uniformly under the cover slip. The motility was recorded using ×200 magnifications with a phase contrast microscope equipped with a thermostatically controlled warm stage. Percent progressive motility (0-100%) was observed at five representative fields of the glass slide. The average of the five scores was recorded. The spermatozoa concentration was measured by hemocytometer chamber method. Briefly, in a known volume of diluted semen sample the number of spermatozoa in counted in a hemocytometer chamber under ×45 magnification and then the total number of spermatozoa is calculated in 1 ml of semen. The percent live spermatozoa, and acrosomal damage was screened by laid down staining technique [24] with a slight modification. Briefly, 10 μl of semen sample was mixed with 100 μl eosin-nigrosine staining solution. 10 μl of the suspension was transferred to a pre-warmed (37°C) slide for preparing a smear. Duplicate smears were prepared from each sample and allowed to air-dry at room temperature. About 200 spermatozoa were observed under ×500 magnifications. The non-eosinophilic (live) and eosinophilic (dead) spermatozoa were counted to calculate the percent live spermatozoa. The same slide was used for screening acrosomal damage. The spermatozoa with intact acrosome without bubbling and swelling were categorized as an undamaged acrosome. Spermatozoa with bubbled and swelled acrosomes, separated acrosomes from sperm head and naked head were considered as I, II and III degree acrosomal damage.

### Statistical analysis

Data of spermatozoa quality attributes of different treatments were analyzed using one-way analysis of variance (SAS, 9.4). Differences among the treatments were determined using *post-hoc* Tukey’s test (SAS, 9.4) and indicated by the superscripts (p*<*0.05). Least squares means standard errors of means and p values were reported. The level of significance was set at p*<*0.05.

## Result

The seminal attributes of fresh ejaculate are presented in Table-1. The seminal attributes of different bulls were statistically similar. The effect of T1 (TE based extender + glutamine) and T2 (50% TE + 50% defatted milk + glutamine) on spermatozoa quality after equilibration is presented in Table-2. Progressive motility and percent live spermatozoa was significantly higher (p<0.05) in T1 as compared to T2 and control. Percent live spermatozoa were significantly higher (p<0.05) in T2 as compared to control. The acrosomal damage decreased (p<0.05) significantly in T2 as compared to T1 and control, and acrosomal damage also decreased significantly in T2 as compared to control. The effect of T1 and T2 on post-thaw spermatozoa quality is presented in Table-3. Progressive motility and percent live spermatozoa was significantly higher (p<0.05) in T1 as compared to T2 and control whereas no significant difference (p>0.05) was observed between control and T2. The acrosomal damage decreased (p<0.05) significantly in T1 as compared to control and T2 whereas it was significantly (p<0.05) lower in T2 as compared to control.

**Table-1:** Seminal attributes of the fresh ejaculate of buffalo bulls used as semen donors.

Animals	Volume (ml)	Mass motility	Concentration (10^9^/ml)	% Live spermatozoa	% Acrosomal damage
Bull no. 3	1.72±0.07	3.27±0.16	0.83±0.008	85.11±0.75	19.46±0.37
Bull no. 5	2.10±0.15	3.11±0.13	0.98±0.15	88.88±0.63	18.95±0.45
Bull no. 9	2.56±0.26	3.44±0.17	0.99±0.01	86.66±0.62	18.81±0.35
Bull no. 10	2.81±0.06	3.22±0.12	0.99±0.10	87.66±0.78	18.95±0.46
Bull no. 11	2.12±0.14	3.11±0.11	0.90±0.14	86.11±0.56	18.62±0.48

**Table-2:** Effect of glutamine supplementation and replacement of TE based extender with defatted cow milk on spermatozoa quality post equilibration.

Parameters	TE (control)	TE+G (T1)	EYT+G+M (T2)	SEM	p-value
Progressive motility	48.88^a^	59.35^b^	52.22^a^	1.30	0.000
Live (%)	67.49^a^	75.20^c^	70.86^b^	0.58	0.000
Acrosomal damage (%)	33.89^b^	32.57^a^	36.03^c^	0.14	0.000
Type 1	22.75^a^	22.54^ab^	23.35^bc^	0.20	0.03
Type 2	6.33^a^	6.06^a^	7.03^b^	0.13	0.000
Type 3	4.81^a^	3.97^b^	5.65^c^	0.20	0.000

Means bearing different superscripts in rows differ significantly (p<0.05). TE based extender (TE), glutamine (G), defatted cow milk (M), Type 1=Bubbled and swelled acrosomes, Type 2=Separated acrosomes from sperm head, Type 3=Naked head, TE=Tris-egg yolk

**Table-3:** Effect of glutamine supplementation and replacement of TE based extender with defatted cow milk on post thaw spermatozoa quality.

Parameters	TE (control)	TE+G (T1)	EYT+G+M (T2)	SEM	p value
Progressive motility	15.91^b^	13.84^a^	16.01^b^	1.54	0.000
Live (%)	57.62^b^	67.20^a^	61.40^b^	2.19	0.003
Acrosomal damage (%)	50.40^c^	48.47^a^	49.83^b^	0.16	0.000
Type 1	21.78^c^	20.41^a^	21.30^b^	0.32	0.004
Type 2	12.71^b^	14.22^a^	12.52^b^	0.32	0.000
Type 3	15.91^b^	13.84^a^	16.01^b^	0.27	0.000

Means bearing different superscripts in rows differ significantly (p<0.05). TE based extender (TE), glutamine (G), defatted cow milk (M). Type 1=Bubbled and swelled acrosomes, Type 2=Separated acrosomes from sperm head, Type 3=Naked head, TE=Tris-egg yolk

## Discussion

The cryopreservation of semen includes the decrease in temperature and increase in oxidative stress on the sperm membrane which results in irreversible damage to intracellular structures and changes in enzymatic activity and associated reduction in motility, fertilizing ability of spermatozoa [25]. Amino acids are the constituents of the seminal plasma and have been used in different combinations in extenders for the cryopreservation of semen in different livestock species [12,18,26] by virtue of their membrane protecting ability. In the present experiment, the supplementation of glutamine to TE based extender improved the spermatozoa quality with respect to the progressive motility, percent live spermatozoa and acrosomal damage both after the equilibration and post-thaw. Although, the mechanism of action of glutamine as a cryoprotectant is still not clear but many authors have reported that glutamine possess the anti-oxidative capacity [12,15,20] which may be attributed to its cryoprotective nature. Supplementation of glutamine to TE based extender increased post-thaw motility pattern and plasma membrane integrity of spermatozoa as compared to control in buffalo bull semen [20]. The addition of glutamine, glycine, and cysteine in the conventional freezing medium enhanced post-thaw motility and improved membrane and acrosome integrity of buffalo bull semen [26]. The result of the present study corroborated with the cryoprotective effects of supplementation of glutamine alone or in combination with other additives in semen extender in different species on post-thaw motility, acrosomal integrity and percent live spermatozoa [15-19].

In the present study, the replacement of 50% TE based extender with defatted cow milk and supplementation of glutamine decreased the post-thaw spermatozoa quality as compared to semen extended with only TE based buffer supplemented with glutamine. However, the former increased the post-thaw spermatozoa quality as compared to control. In the present study, the post-thaw motility, acrosomal integrity, and percentage of live spermatozoa was marginally better in T2 because of supplementation of glutamine. The results indicated that defatted cow milk was not as effective as TE based diluent having the same additives. Milk based extenders are reported to be equally effective as egg yolk based extenders [27]. The previous studies suggested that lipid portion of the milk was not responsible for protecting the spermatozoa stored at 4°C [28] and it was demonstrated that milk proteins were of cryoprotective nature [29]. On contrary to previous reports, the present study revealed that the protective effect of defatted cow milk in comparison to TE was not appreciable.

## Conclusion

From the results of present study, it can be concluded that glutamine supplementation was effective in maintaining post-equilibration and post-thaw spermatozoa quality whereas defatted cow milk was not as effective as TE based buffer in the extender in improving the spermatozoa quality.

## Authors’ Contributions

SY designed the experimental program. VD carried out the experiment. BY analyzed the data and prepared the manuscript. All authors read and approved the final manuscript.
